# Physicochemical and Functional Properties of Black Walnut and Sycamore Syrups

**DOI:** 10.3390/foods13172780

**Published:** 2024-08-31

**Authors:** Olivia McHugh, Elijah Ayilaran, Anthony DeBastiani, Yangjin Jung

**Affiliations:** 1 Agricultural & Environmental Research Station, West Virginia State University, Institute, WV 25112, USA; olivia.mchugh@wvstateu.edu; 2 Department of Biology, West Virginia State University, Institute, WV 25112, USA; eayilaran@wvstateu.edu; 3 Shared Research Facilities, West Virginia University, Morgantown, WV 26505, USA; apd0006@mail.wvu.edu

**Keywords:** black walnut syrup, sycamore syrup, physicochemical, antioxidant activity, phenolics, antimicrobial activity

## Abstract

Historically, tree sap has been used globally for medicinal purposes, in fermented beverages, and for syrup production. Maple tree sap is notably concentrated into syrup and is valued as a natural sweetener rich in phenolic compounds and minerals compared to refined sugar. Recently, syrups from other trees like black walnut (*Juglans nigra*) and sycamore (*Platanus occidentalis*) have gained popularity, yet their properties are not well understood scientifically. To address this gap, we collected sycamore, black walnut, and maple syrup samples and analyzed their physicochemical and functional properties. Our findings showed significant differences among the syrups in pH, browning intensity, and water activity (*p* < 0.05). Sycamore syrup had the highest total phenolic content, followed by black walnut and maple syrups. Both black walnut and sycamore syrups exhibited similar antioxidant activity, significantly higher than maple syrup (*p* < 0.05). High-resolution mass spectrometry identified 54 phenolic acids and 22 flavonoids in these syrups, including Acetylsalicylic acid, 3,5-Dihydroxybenzoic acid, and syringic acid, known for their antioxidant and anti-inflammatory properties. Additionally, sycamore syrups and most black walnut syrups displayed varying degrees of antimicrobial activity against Gram-positive and/or Gram-negative microorganisms. This study offers insights into the properties and potential health benefits of these specialty tree syrups.

## 1. Introduction

Excessive intake of processed/refined sugars (e.g., white sugar and high-fructose corn syrup) is a major risk factor for several diseases, such as obesity, diabetes mellitus, metabolic syndrome, and cardiovascular diseases. To address the prevalence of these conditions linked to added sugars, extensive research has focused on developing natural alternative sweeteners such as nipa palm syrup [1], date palm syrup [2,3], sweet sorghum syrup [4], agave syrup [5], and maple syrups [6].

Tree sap has been utilized for diverse purposes in numerous countries, serving medicinal, fermented beverage, and syrup uses throughout history [7]. North American Native communities have been tapping maple trees since time immemorial; maple sap and syrup are integral to Iroquois origin stories [8]. Birch sap tapping has a longstanding tradition in European countries, and upon European settlement in America, the tapping technique was refined on maple trees. As a result, maple syrup from North America has become the most renowned variety [7,9]. While maple syrup is a representative refined sugar alternative sweetener derived from tree sap [6,10], other natural sweeteners from tree sap, such as black walnut (*Juglans nigra*), birch (*Betula* spp.), sycamore (*Platanus occidentalis*), butternut (*Juglans cinerea*), and others have gained popularity among consumers, thanks in part to the rise of direct market and e-commerce platforms. These marketplaces have empowered small-scale agricultural producers, enabling them to easily reach a broader audience and fostering a growing trend that grants consumers convenient access to a diverse range of gourmet products [11].

Black walnut (*Juglans nigra*), also known as eastern black walnut, and sycamore (*Platanus occidentalis*) species thrive throughout the central and eastern parts of the United States. According to Naughton et al. [9], there was an absence of a tapping site for commercial walnut syrup production in 2006 in the United States. However, recent developments have led to the availability of black walnut syrup in local farmers’ markets and online venues. The production of marketable sycamore syrup is more novel, and there is currently a lack of commercial availability. It was reported that the average price per gallon of maple syrup in the United States was USD 37.50 in 2022 [12], while specialty syrups like black walnut and sycamore fetched significantly higher prices ranging from USD 150 to USD 350 per gallon [13]. Moreover, Collins-Simmons and her colleagues highlighted that exploring the marketability of sycamore syrup could enhance the economic value of these trees [13]. Sycamore trees, traditionally classified as low-value hardwoods, play a crucial role in maintaining riparian zones to prevent flooding, controlling erosion of stream banks, and supporting the local ecosystem [13]. Despite the conventional understanding that black walnut sap flow is less than that of maple, a field study conducted by Cornell University [14] revealed that, with the use of a vacuum pump, black walnut sap yield could be comparable to maple. Similarly, another field trial from Future Generations University showed that a vacuum pump also resulted in an increase in the level of sap collected from sycamore trees [15]. This suggests promising economic potential from both tree types that has yet to be fully investigated.

Unlike maple syrup, extensively studied for its biological effects such as antioxidant, anti-inflammatory, anticancer, antidiabetic, and neuroprotective properties [6,16,17,18], there remains a significant gap in the scientifically established understanding of the physicochemical and functional properties of sycamore and black walnut syrups. To the best of our knowledge, no studies have been conducted on these properties. Understanding the physicochemical properties of syrups provides crucial scientific data necessary for ensuring quality control, optimizing processing conditions, and utilizing these syrups in value-added products. Furthermore, exploring the potential health benefits of these syrups promotes value-added agriculture, supports economic development, and enhances resilience in rural communities. Consumers can access healthier food options derived from advanced scientific knowledge.

Therefore, this research aims to investigate and determine the physicochemical, antioxidant, and antimicrobial properties of black walnut and sycamore syrups produced in the eastern United States. By addressing this research gap, we aim to provide valuable insights into the potential health benefits and marketable properties of specialty tree syrups, thereby enhancing their position in the natural sweetener market.

## 2. Materials and Methods

### 2.1. Syrup Collection

Syrup samples were obtained from multiple syrup producers from Missouri, Ohio, New York, Virginia, and West Virginia from September 2023 to May 2024. The collected syrups are listed in Table 1, which includes both light- and dark-grade maple syrups purchased for comparison purposes.

### 2.2. Physicochemical Characteristics

The water activity (Aw) of each sample was assessed utilizing a dew point sensor in an AquaLab Series 4TE water activity meter (Decagon Devices Inc., Pullman, WA, USA). The instrument underwent calibration with a 0.760 aw 6.00 mol/kg NaCl verification solution. Each sample was half-filled into a disposable sample cup (Decagon Devices Inc., Pullman, WA, USA) and positioned in the Aw meter chamber. The measurement temperature was set to 20 °C. The pH measurements were conducted on undiluted syrup samples using a pH meter with a Sure-Flow epoxy-body pH electrode (Orion Star A211, Thermo Fisher Scientific, Waltham, MA, USA) following calibration. Total Soluble Solids (TSS) were determined at room temperature employing a Digital Handheld Refractometer (VWR International, Leuven, Belgium), and the results were recorded in Brix (°Bx). Density was measured at room temperature using a 10 mL glass calibrated pycnometer (Eisco™, Honeoye Falls, NY, USA). The browning intensity of syrup samples was assessed by measuring absorbance at 420 nm with a spectrophotometer (UV-1600PC, VWR International, Leuven, Belgium). Samples were appropriately diluted to a 1% solution in deionized water to achieve an optical density of less than 1.5 [19]. The color values of the syrups, including lightness (L*), redness–greenness (a*), and yellowness–blueness (b*), were measured using a portable colorimeter equipped with a cell holder and plastic cell (10 mm CM-A131) (Chroma Meter CR-400, Konica Minolta, Ramsey, NJ, USA).

### 2.3. Total Phenolic Content

Total phenolic content (TPC) was measured using the Folin–Ciocalteu method described by Thabet et al. [20] and Asghar et al. [21] with some modifications. Ten grams of each syrup sample was mixed with 25 mL of a 50% (*v*:*v*) methanol/water solution and shaken for 30 min. After shaking, the solution was filtered through a Whatman no.1 filter(Cytiva, Marlborough, MA, USA), and the filtrate was used to perform the assay. The solution was further diluted with deionized water for some of the samples due to their dark color in order to achieve an absorbance value within the standard curve. Ten microliters of prepared sample solution, standard solutions, or deionized water to be used as a blank was pipetted into individual wells of a 96-well plate. This was followed by the addition of 100 µL of 1:10 water diluted Folin–Ciocalteu reagent (Supelco^®^, Bellefonte, PA, USA) to each well. The plate was then shaken for 1 min. Eighty microliters of a 6% Sodium carbonate (Na_2_CO_3_) solution was then added to each well, and the plate was left to stand in the dark at room temperature for 90 min. After incubation, absorbance was read at 700 nm using an Accuris SmartReader 96 (Accuris Instruments, Edison, NJ, USA). Gallic acid (3,4,5-trihydroxybenzoic acid) was used as a spectrophotometric standard and was prepared in dilutions of 100–1000 µg/mL in deionized water. The standard curve was used to calculate gallic acid equivalents of the samples, and total phenolic content was calculated using the following equation adapted from Kaškonienė et al. [22]:

*C* = *c* × *m*_1_/*m*_2_ (1)

where *C* is the total phenolic content in µg/mL of syrup, *c* is the concentration of gallic acid equivalent (µg/mL) established from the standard curve (*r*^2^ = 0.9647–0.999), *m*_1_ is the weight of syrup solution (g), and *m*_2_ is the weight of syrup (g). Samples that were diluted due to darker color were adjusted by multiplying with the appropriate dilution factor.

### 2.4. Antioxidant Activity

Antioxidant activity was determined using the 2,2-diphenyl-1-picrylhydrazyl (DPPH) radical scavenging activity method according to Ben Thabet et al. [20] with minor modifications. One gram of syrup was mixed with 5 mL of deionized water and then filtered through a Whatman no. 1 filter(Cytiva, Marlborough, MA, USA). The filtrate was diluted with deionized water to 4 º Bx using a handheld refractometer (VWR)(VWR International, Radnor, PA, USA). One syrup sample, Maple Syrup 1, was diluted in 2 mL of deionized water rather than 5 mL. This adjustment was made to achieve a sufficient absorbance for measurement at 520 nm. Fifty microliters of samples was added to individual wells of a 96-well plate. Methanol was used as a blank, and deionized water was used as a negative control. An amount of 195 µL of 0.1 mM DPPH in methanol was added to each well except the blank. The plate was incubated in the dark at room temperature, and absorbance was read after 60 min at a wavelength of 520 nm using an Accuris SmartReader 96 (Accuris Instruments, Edison, NJ, USA). Antioxidant activity is expressed as the percentage of inhibition of the DPPH radical, calculated by the following equation:

% Antioxidant Activity = [(*A*_1_ − *A*_2_)/*A*_1_] × 100 (2)

where *A*_1_ is the absorbance of the negative control reaction, and *A*_2_ is the absorbance of the reaction, including the sample.

### 2.5. Antimicrobial Activity

The antimicrobial activity of the syrup was analyzed using the well diffusion method according to Oboh et al. [23] with some modifications. *Escherichia coli* ATCC 25922, *Pseudomonas aeruginosa* ATCC 27853, and *Staphylococcus aureus* ATCC 25923 were purchased from MicroBiologics (KWIK-STIK™, St. Cloud, MN, USA). Freshly grown bacterial culture was adjusted to McFarland Standard No 0.5 (Hardy Diagnostics, Santa Rosa, CA, USA) and used to swab the entire surface of solidified Mueller Hinton Agar plates (Difco, BD, Sparks, MD, USA). Three holes were made aseptically in each plate using a 9.5 mm sterile cork borer(Humboldt Manufacturing, Chicago, IL, USA). In one well per plate, 100 µL of each pure syrup sample was added. One hundred microliters of sterile deionized water was added to a second well as a negative control, and Streptomycin sulfate (Sigma-Aldrich, St. Louis, MO, USA) at a concentration of 130 mg/L was added to the third well as a positive control. Plates were incubated at 37 °C for 24 h, followed by measurement of inhibition zones surrounding the wells to indicate the degree of sensitivity of the test organism to each sample.

### 2.6. Non-Targeted Metabolomic Analysis

Samples were prepared by diluting a 1 mL aliquot of maple, black walnut, or sycamore syrup 1:1 with methanol (Optima LC-MS grade, Fisher Scientific, Pittsburgh, PA, USA) in a plastic centrifuge tube. Samples were vortexed for 30 s and centrifuged at 13,400 rcf for 20 min at 4 °C to precipitate undissolved material. An amount of 200 µL of supernatant was diluted to 1 mL with methanol, mixed, and filtered through a 0.22 µm PVDF syringe filter (VWR, Visalia, CA, USA) into a glass HPLC vial for analysis. Pooled quality control (QC) reference samples were prepared utilizing an equal amount of each sample included and subjecting it to extraction.

Non-targeted metabolomic analysis of each sample was conducted utilizing a Thermo Fisher Q-Exactive quadrupole-orbitrap high-resolution mass spectrometer (HRMS) coupled to a Vanquish ultra-high-pressure liquid chromatography (UHPLC) system (Thermo Fisher Scientific Inc., San Jose, CA, USA). Analytes were separated utilizing a Waters Acquity UPLC BEH Amide column, 1.7 µm, 2.1 mm × 50 mm (Waters Corporation, Milford, MA, USA) maintained at a temperature of 35 °C via gradient elution using LC-MS grade water with 0.1% formic acid (A) and a mixture of acetonitrile (ACN) (Optima LC-MS grade, Fisher Scientific, Pittsburgh, PA, USA) and water (90:10, *v*/*v*) with 0.1% formic acid (B). The flow rate was set to 150 µL/min with the gradient elution program as follows: 0–2 min hold at 100% B, 2–18 min linear gradient 100–40% B, 18–20 min hold at 40% B, 20–21 min linear gradient 40–100% B, 21–23 min hold at 100% B. Mass spectrometry instrument conditions for both positive and negative ionization modes were optimized using the auto defaults in the X-calibur software(version 4.4) for a flow rate of 150 µL/min: capillary temperature 400.0 °C, heater temperature 412.5 °C, sheath gas flow rate 45 arb (arbitrary units), auxiliary gas flow rate 15 arb, sweep gas flow rate 2.25 arb, S-lens voltage of 50 V, and spray voltages of 4.0 kV (positive) and 3.0 kV (negative). Full scan mode was utilized with a scan range of 70 to 1000 mass-to-charge (*m*/*z*) with a resolving power of 70,000 and an automatic gain control (AGC) target of 1 × 10^6^ with an injection time (IT) of 50 ms.

Mass spectrometry data were collected for each sample, and raw data were imported into Compound Discoverer 3.2 software (Thermo Fisher Scientific Inc., San Jose, CA, USA). A workflow was created to identify unknown compounds present. The possible chemical composition of the samples was obtained through primary matching and screening utilizing the ChemSpider database with a further comparison of secondary fragment ions generated during high-energy collisions within the mass spectrometer using the mzCloud and mzVault databases. Obtained compound lists were visually inspected and filtered to include peaks with a signal-to-noise ratio (S/N) of at least 1.5, a matching value of over 80, and *m*/*z* width of not more than 5 ppm. Following the processing of the data, compound lists were exported into an Excel spreadsheet for further evaluation, and relative normalized responses for compounds of interest were obtained utilizing a pooled reference sample for each tree type.

### 2.7. Statistical Analysis

All experiments were repeated independently in triplicate. Data are expressed as mean ± standard deviation values between black walnut (*n* = 7), sycamore (*n* = 8), and maple (*n* = 4) syrups. Data were analyzed using one-way analysis of variance, and a comparison of means was carried out using Duncan’s multiple-range test to identify significant differences (*p <* 0.05) between syrup types using Statistical Analysis System (SAS^®^ Studio 3.81 for Windows, SAS Institute Inc., Cary, NC, USA).

## 3. Results and Discussion

### 3.1. Physicochemical Properties of Syrups

Table 1 presents the physicochemical properties of collected syrups. In accordance with the Code of Federal Regulation Title 21, section 168.140, maple syrup derived by concentration and heat treatment of maple tree (Acer) sap must contain no less than 66% by weight of soluble solids, or 66 °Bx, and must not include added sweeteners [24]. Total soluble solids between different types of syrup were measured in °Brix, and while the °Brix values of two out of eight black walnut syrups, one maple syrup, and one sycamore syrup were below 66 °Brix, all other collected samples exceeded this specified criterion. The average °Brix values for black walnut, maple, and sycamore syrups were 67.4 ± 1.9, 66.3 ± 1.6, and 68.5 ± 2.29, respectively. There was no significant difference in total soluble solid content between the three syrup types tested (*p* > 0.05).

The average pH values among all syrup types varied significantly (*p* < 0.05) (Table 1). The pH of black walnut syrups ranged from 4.6 to 5.3 with an average of 5.04, and maple syrups exhibited a range of 6.2 to 7.0 with an average of 6.6, while sycamore syrups displayed a pH range of 3.9 to 4.2 with an average of 4.10, which defines them as acid foods. Acid foods are more shelf stable as most microorganisms are unable to grow in the presence of an acidic pH [25]. The acidic pH likely resulted from the evaporation of moisture during heating, causing an elevation in the concentration of organic acids that were present in the sap. Moreover, the formation of certain organic acids can occur through the Maillard reaction during the development of the brown color, as documented by Ho et al. [26] and Lertittikul et al. [26].

The formation of the brown color in syrup is attributed to non-enzymatic Maillard browning reactions during the thermal processing of tree sap, which play a crucial role in determining both the color and flavor of the syrup and are responsible for the formation of antioxidant compounds [27,28,29].

The degree to which these reactions occurred during thermal processing can be measured by analyzing the absorbance of a 1% solution of the sample at a wavelength of 420 nm to measure browning intensity. As seen in Table 2, the average browning intensity of maple syrup was 0.028, while black walnut syrups averaged 0.229 and sycamore syrups averaged 0.417. Black walnut and sycamore syrups did not have a significant difference in their browning intensities, nor did maple and black walnut syrups; however, sycamore and maple syrup samples were significantly different (*p* < 0.05). This indicates that sycamore syrup has more Maillard reaction products than maple, which likely has an influence on its sensory attributes and increases its antioxidant potential. The color values for maple, black walnut, and sycamore samples, including lightness (L*), redness–greenness (a*), and yellowness–blueness (b*) are indicated in Table 2. For black walnut syrup samples, the L* values ranged from 17.6 to 18.09, a* values ranged from 0.36 to 2.01, and b* values ranged from 0.8 to 1.16. L* values for sycamore syrups ranged from 17.75 to 24.22, a* values from 0.47 to 17.56, and b* values from 0.8 to 11.73. For maple syrup samples, 19.51–55.53, 0.19–17.8, and 3.62–36.16 were the ranges for L*, a*, and b* values, respectively. Indeed, the color of maple syrup is influenced by factors such as its origin, microbial load, and heat treatment, ultimately impacting its grade, with lighter-colored syrups being more expensive [29]. While there is no classification guide for colors of black walnut and sycamore syrups, maple syrups suitable for sale are classified by the USDA into four categories: US Grade A Golden, Amber, Dark, and Very Dark based upon percent light transmittance [30]. Flavors become stronger and more robust as color gets darker. Historically, consumers preferred the taste of the lightest color, which offers a subtle maple and buttery flavor; however, recently, more consumers have been gravitating toward the more full-bodied flavors of darker syrups [31]. One consumer analysis by Matta et al. [32] compared the preference consumers had between maple syrup and black walnut syrup and found no significant difference in which type of syrup they preferred. While there is not currently any data on the consumer preference for sycamore syrup, some customers may prefer the darker color that is observed in sycamore and black walnut syrups, which is associated with a stronger and more flavorful product.

The water activity (Aw) of black walnut syrups ranged from 0.8090 to 0.8477, which is significantly higher (*p* < 0.05) than that of sycamore syrups (0.7249–0.7512) but remains comparable to maple syrups (0.8451–0.8715) (Table 1). This could indicate that sycamore syrups are less likely to tolerate microbial growth and are more shelf-stable than black walnut and maple syrups, as lower water activity is indicative of less moisture available for the reproduction and biochemical processes of most microorganisms [33]. The density of syrups was fairly consistent within the range of 1.32 to 1.36 g/mL among all collected samples, with no significant difference in the mean density between syrup types (*p* > 0.05). Notably, limited research has been conducted on the physicochemical properties of black walnut and sycamore syrups, with no studies having been conducted on these properties for sycamore syrup and only one study by Naughton et al. [9] providing some insights into this aspect for black walnut syrups (noted as a non-commercial product). According to Naughton et al. [9], the black walnut syrup exhibited pH, Aw, and total soluble solids values of 6.75, 0.858, and 65.1%, respectively. In their study, values for maple syrup were reported as 6.88, 0.844, and 68.2%, providing a basis for comparison.

### 3.2. Total Phenolic Content

**Figure 1:**
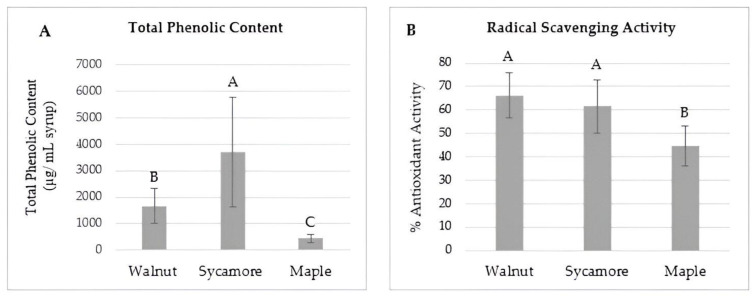
Total phenolic content and antioxidant (DPPH radical scavenging) activity of specialty tree syrups: (**A**) Total phenolic content expressed as µg/mL syrup. (**B**) Radical scavenging activity expressed as % antioxidant activity. Values are expressed as mean between samples of black walnut (*n* = 7), sycamore (*n* = 8), and maple (*n* = 4) syrups. Error bars show standard deviation for each mean value; different letters within each graph represent statistical differences (*p* < 0.05).

Phenolic compounds are a diverse group of bioactive substances found in many plant foods that contribute to sensory properties associated with food, such as color, flavor, and aroma, and have been researched extensively in recent years due to their potential health benefits [20,34]. Many polyphenols have been shown to express antioxidative, anti-inflammatory, and antiallergic properties [35]. They also are known to act as antimutagens, anticarcinogens, and antimicrobial agents [23]. It has been shown that phenolic compounds are a predominant component in maple syrup [6] and are partially responsible for its distinct flavor [29]. However, there is currently a lack of scientific research on the presence of polyphenols in black walnut and sycamore syrups. To address this gap in the knowledge, the total phenolic content for maple, black walnut, and sycamore syrup samples was determined using the Folin–Ciocalteu assay. The results are expressed as averages of total phenolic content (µg/mL syrup) for each syrup type in Figure 1A. The average total phenolic content values for black walnut, sycamore, and maple syrups were 1669.47 ± 653.29, 3968.53 ± 1747.03, and 439.82 ± 161.50 µg/mL syrup, respectively. Black walnut syrups demonstrated a significantly higher total phenolic content than maple syrups (*p* < 0.05), and sycamore syrups had a significantly higher phenolic content than both walnut and maple syrups (*p* < 0.05). It is important to note that the Folin–Ciocalteu reagent is intended to react with phenolic products to measure total phenolic content; however, the reagent is also known to react with additional products likely present in the samples including reducing agents and Maillard reaction products, such as fructose and melanoidins [36]. These off-target reactions could affect the accuracy of the measurement of total phenolic content. Eggleston et al. [4] evaluated the phenolic contents and antioxidant potential of sweet sorghum syrups compared to maple and other syrups. They found that 10 sweet sorghum syrups contained 6471 ± 1823 mg/L of phenolic compounds, whereas three maple syrups showed less than 200 mg/L, which aligns with findings from the current study on maple syrup. Thériault and colleagues [35] determined the total phenolic compounds of maple syrup. The gallic acid equivalent (GAE) quantity of the total phenolic compounds in the syrups ranged from 17.81 to 63.81 g GAE/100 g, varying by season. Since phenolic compounds are well known to express many bioactivities and properties that are beneficial to human health and are known to contribute to the flavor profile of other tree syrups, these data could provide a basis for future investigations into potential health benefits attributable to the consumption of black walnut and sycamore syrups, along with insights into how these compounds affect the flavor and consumers’ preference of specialty syrups.

### 3.3. Antioxidant Activity

Free radicals are byproducts produced during many biological processes and can also be encountered exogenously from sources such as pollution, medication, and radiation [37]. Accumulation of free radicals in the body can lead to oxidative stress and various degenerative disorders, including autoimmune disease, cancer, and neurodegenerative and cardiovascular issues [37]. Antioxidants are compounds that occur naturally in many foods and are responsible for protecting against oxidative damage from free radicals in the body [38]. Phenolic compounds present in maple sap and syrup have been shown to exhibit antioxidant and antiradical activities through mechanisms such as scavenging of free radicals, quenching of reactive oxygen species, or inhibiting oxidative enzymes [20,35]. Therefore, the antioxidant activity of black walnut, sycamore, and maple syrups was evaluated using the DPPH radical scavenging assay [20], in which the free radical scavenging capacity of antioxidants within the sample is measured spectrophotometrically as a percentage of inhibition of the DPPH free radical. The average antioxidant activities of black walnut, sycamore, and maple samples were 66.11 ± 9.65%, 61.46 ± 11.2%, and 44.5 ± 8.62%, respectively. Both black walnut and sycamore syrup types exhibited significantly higher (*p* < 0.05) antioxidant activity levels than maple syrups, and there was no significant difference between black walnut and sycamore syrups (Figure 1B).

Likely contributing to the radical scavenging ability of the syrups are Maillard reaction products, which are formed through a non-enzymatic reaction between reducing sugars and amino acids, peptides, or proteins upon heating and are known to possess antioxidant potential [39]. In addition to this, significant positive correlations between antioxidant activity and total phenolic content have been reported [40], as the majority of phenolic compounds have the potential to perform antioxidant activities in some capacity. This is supported by our data as walnut and sycamore syrups both demonstrated significantly higher total phenolic content and antioxidant activity than maple. While there is no published data on the composition of the phenolic compounds present in black walnut and sycamore syrups, it is known that the phenolic content of maple syrup is composed of many phenolic compounds, including lignans, coumarins, stilbenes, and phenolic derivatives, all of which have varying levels of antioxidant activity [41]. The antioxidant activity of phenolic compounds in maple syrup has also been shown to vary throughout the season, depending on harvesting time [35]. It should also be noted that the ability of different Maillard reaction products to scavenge free radicals is dependent upon several variables, including the type of reducing sugars or amino acids used as substrates in the reaction, as well as the time and intensity of heating [42]. The variability in the composition of phenolic compounds within the different syrup types, the differences in the presence and antioxidant capacity of Maillard reaction products, and the off-target effects of these products reacting with the Folin–Ciocalteu reagent during measurement of total phenolic content could all contribute to walnut syrup demonstrating significantly higher total phenolic content yet similar antioxidant activity to sycamore syrup. Overall, the significantly higher antioxidant activity of walnut and sycamore syrups in comparison to maple indicates that the phenolic compounds and Maillard reaction products present in these samples have a greater capacity to scavenge harmful free radicals, further validating the potential for health benefits of these specialty tree syrups that remain to be investigated.

### 3.4. Antimicrobial Properties

The antimicrobial activities of various syrup samples were investigated against *Escherichia coli* (*E. coli*), *Pseudomonas aeruginosa (P. aeruginosa*), and *Staphylococcus aureus* (*S. aureus*) using the well diffusion assay method. Gram-negative bacteria such as *P. aeruginosa* and *E. coli* have an outer membrane containing lipopolysaccharides and a thinner peptidoglycan layer, contributing to antibiotic resistance and leading to their association with respiratory and urinary tract infections [43]. Gram-positive bacteria, such as *S. aureus*, are characterized by their thick peptidoglycan layer and lack of an outer membrane, often associated with diseases of the skin and soft tissues [44,45]. By assessing the antimicrobial activity of syrup samples against these bacterial types, this study aims to explore their broad-spectrum antimicrobial potential.

As seen in Table 3, sycamore syrups exhibited the most significant antimicrobial activity, with seven out of eight samples creating inhibition zones against *S. aureus*, five samples demonstrating antimicrobial activity against *P. aeruginosa*, and four samples against *E. coli*. Black walnut syrups exhibited moderate activity, with two samples showing considerable inhibition of *S. aureus* growth and three showing light antimicrobial activity against *P. aeruginosa*. Maple syrup samples did not show any antimicrobial activity against any of the tested bacterial strains. However, Maisuria et al. [46] extracted phenolic compounds from maple syrup (PRMSE) and demonstrated their antimicrobial and antibiofilm effects against pathogenic bacteria, including *E. coli* strain CFT073 (ATCC 700928), *P. mirabilis* HI4320 (17), *P. aeruginosa* PAO1 (ATCC 15692), and *P. aeruginosa* PA14 (UCBPP-PA14). PRMSE enhances antibiotic susceptibility in both planktonic and biofilm growth, potentially by permeabilizing bacterial membranes, inhibiting multidrug resistance pumps, and downregulating multidrug resistance genes. Catechol, one of the tested phenolics, plays a crucial role in PRMSE’s synergy with an antibiotic (ciprofloxacin). It is important to note that the difference in results may be attributed to variations in target strains and the concentration of the active compounds. The current study tested the syrup itself, whereas Maisuria et al. [46] evaluated a polyphenolic extract from maple syrup.

The results indicate that sycamore syrups and black walnut syrups exhibit antimicrobial effects, which is unsurprising given that prior research has shown that sycamore leaves contain compounds effective against methicillin-resistant *S. aureus* (MRSA) and other pathogens [47]. Additionally, walnut husk contains naphthoquinones, particularly juglone, 1,4-naphthoquinone, and plumbagin, which have been shown to exhibit inhibitory effects on the growth of various bacteria, including *B. cereus, B. subtilis, and P. aeruginosa* [48]. Notably, syrups from black walnuts prevented the growth of both *S. aureus* and *P. aeruginosa*. This observation aligns with the literature, indicating that certain components of black walnuts possess antibacterial properties, particularly against *S. aureus* [49]. The antimicrobial properties of sycamore syrups can be partially attributed to flavonoids known to inhibit both Gram-positive and Gram-negative bacteria [50]. The high phenolic content in sycamore syrups likely enhances their antimicrobial effectiveness, as plant-derived phenolics have demonstrated inhibitory effects on microbial growth [51]. The resistance shown by *E. coli* shows the intricate nature of microbial interactions and suggests that the antimicrobial compounds in the syrups may exert specific activities.

Therefore, further research into the specific antimicrobial compounds of those syrups and their mode of action, as well as exploring potential applications of sycamore syrups as natural antimicrobial agents, is warranted.

### 3.5. Phenolic and Flavonoid Compound Profiles

The phenolic and flavonoid profiles of maple, black walnut, and sycamore syrups were identified using Quadrupole Orbitrap High-Resolution Mass Spectrometry in a non-targeted metabolomics approach. Analysis with Compound Discoverer software (3.0)revealed a total of 169 compounds in negative ion mode and 386 in positive ion mode. These compounds encompassed a wide range of metabolites, including amino acids, carbohydrates, flavonoids, catechols, and other plant-derived compounds. Specifically, 54 phenolic acids and 22 flavonoids were identified and detailed in Table S1. This table provides comprehensive information on each annotated compound, including compound names, molecular formulas, classifications, calculated masses, exact masses, retention times, intensity, and relative abundances. Relative abundances were determined using pooled qualitative reference samples of maple, black walnut, and sycamore syrups.

Phenolic acids predominantly appeared in negative ionization mode, while flavonoids were observed in positive ionization mode. Notably, 3,5-Dihydroxybenzoic acid (Gentisic acid), a derivative of salicylic acid known for its antioxidant properties, was prominent in maple and sycamore syrups [52]. Acetylsalicylic acid (Aspirin), recognized for its anti-inflammatory and analgesic effects, was found in sycamore syrup [53]. Syringic acid, noted for antioxidant and anti-inflammatory properties, was present in sycamore and black walnut syrups [54]. Vanillyl mandelic acid, a bioactive compound with antioxidant properties, was identified in walnut syrup [55]. 3,4,5-trihydroxycyclohex-1-ene-1-carboxylic acid (Shikimic acid), known for its pharmacological effects, including anti-inflammatory, antibacterial, and antioxidant properties, was found in sycamore syrup [56]. Quinic acid, present in maple and sycamore syrups, exhibited diverse activities such as antioxidant, antidiabetic, anticancer, antimicrobial, antiviral, and analgesic effects [57]. Benzoylpropionic acid in sycamore is recognized for its anti-inflammatory activity [58,59].

Previous studies have also reported on phenolic compounds in maple products, highlighting substances such as HMF, ferulic acid, vanillin, and syringaldehyde. Additionally, various benzoic acid derivatives, including vanillic acid and syringic acid, as well as cinnamic acid derivatives like p-coumaric acid, ferulic acid, and sinapic acid, have been identified by High-performance Liquid Chromatography (HPLC) [34]. Li and Seeram [41] isolated 23 phenolic compounds from a butanol extract of Canadian maple syrup using chromatography. They further identified these compounds using nuclear magnetic resonance and mass spectral data, which included lyoniresinol, secoisolariciresinol, scopoletin, vanillin, syringaldehyde, gallic acid, syringic acid, syringenin, coniferol, catechol, and others.

This non-targeted metabolomics approach offers a thorough exploration of the phenolic and flavonoid compositions found within maple, black walnut, and sycamore syrups. This analysis underscores the diversity of bioactive compounds present in these natural products and emphasizes their potential health benefits. Moreover, further research is necessary to optimize extraction methods from these syrups and evaluate their pharmacological potential for therapeutic applications, encompassing efficacy, safety, and the specific mechanisms of action of these bioactive compounds across diverse health contexts.

One limitation of this study is that we collected the samples as syrup, making it hard to determine whether the differences seen in the functional properties of the syrups are due to differences in the chemical compositions and biological activities of the compounds in the sap, the processing methods for making the syrup, or a combination of both. Black walnut and sycamore sap are generally processed in the same way as maple sap, through boiling to evaporate water until the desired syrup consistency is achieved. However, while maple and sycamore sap are typically first passed through a Reverse Osmosis (RO) system in order to concentrate prior to heating and reduce boiling time, walnut sap is unable to filter through the RO system due to the presence of what is described as a “pectin-like” substance within the sap [15,60]. The composition of this substance has yet to be determined, although its presence or effect on processing methods could be partially responsible for the differences seen between the syrup types. Controlled studies evaluating the composition and properties of each sap with comparison to syrups produced upon different processing methods are needed to further elucidate the differences in physicochemical and functional properties observed between syrup types.

## 4. Conclusions

This study revealed novel insights into the physicochemical and functional properties of two specialty tree syrups, black walnut (*Juglans nigra*) and sycamore (*Platanus occidentalis*), which are emerging components of the natural sweetener market. The results illustrated significant differences in the pH and water activity of black walnut and sycamore syrups in comparison to the more common and widely studied maple syrup, which could have an impact on the shelf life and antimicrobial capabilities of the syrups. Color values and browning intensity were also analyzed for the first time in sycamore and walnut syrup types, providing a basis for comparison in future studies. Significantly higher total phenolic content and antioxidant activity were demonstrated by the black walnut and sycamore syrups when compared to maple, indicating a potential for health benefits as a result of their consumption. Antimicrobial activity against both Gram-positive and Gram-negative microorganisms was found in sycamore and black walnut syrups, suggesting they may be beneficial in food preservation or for medicinal purposes. To the best of our knowledge, this study is the first to report on the functional properties of black walnut and sycamore syrups. However, due to the limited commercial availability of these syrups, the sample size was constrained. Nevertheless, these scientific data highlight their functional properties and could stimulate interest in their market expansion. Further studies are warranted to explore their biological effects and potential for in vivo applications.

## Figures and Tables

**Table 1:** Physicochemical properties of samples of black walnut, maple, and sycamore syrups.

Sample Code	Syrup Type	Produced in	pH		TSS (°Brx)		Water Activity		Density (g/mL)	
WS 1	Walnut	WV	5.0	* 5.0 ± 0.24 ^B^	68.4	* 67.4 ± 1.9 ^A^	0.8245	* 0.8330 ± 0.01 ^A^	1.34	* 1.34 ± 0.01 ^A^
WS 2	Walnut	WV	5.2		68.4		0.8249		1.34	
WS 3	Walnut	VA	4.6		67.8		0.8465		1.34	
WS 4	Walnut	VA	5.3		66.1		0.8384		1.33	
WS 5	Walnut	MO	5.1		70.4		0.8090		1.35	
WS 6	Walnut	OH	5.2		65.7		0.8397		1.33	
WS 7	Walnut	NY	4.9		64.9		0.8477		1.32	
MS 1	Maple, Golden	WV	6.9	* 6.6 ± 0.41 ^A^	66.4	* 66.3 ± 1.6 ^A^	0.8478	* 0.8533 ± 0.01 ^A^	1.34	* 1.33 ± 0.01 ^A^
MS 2	Maple, Amber	WV	7.0		67.5		0.8451		1.34	
MS 3	Maple, Amber	NY	6.3		64.0		0.8715		1.31	
MS 4	Maple, Dark	NY	6.2		67.3		0.8489		1.33	
SS 1	Sycamore	WV	4.2	* 4.10 ± 0.16 ^C^	69.3	* 68.5 ± 2.29 ^A^	0.7340	* 0.7625 ± 0.03 ^B^	1.35	* 1.34 ± 0.01 ^A^
SS 2	Sycamore	WV	4.4		71.1		0.7249		1.36	
SS 3	Sycamore	WV	4.1		70.2		0.7548		1.35	
SS 4	Sycamore	WV	4.0		68.8		0.7512		1.34	
SS 5	Sycamore	WV	4.0		70.8		0.7385		1.35	
SS 6	Sycamore	WV	4.0		66.3		0.8010		1.33	
SS 7	Sycamore	WV	4.0		65.0		0.8038		1.32	
SS 8	Sycamore	WV	3.9		66.5		0.7921		1.34	

* Indicates mean ± standard deviation value between syrup samples. Different uppercase letters (A–C) in each column indicate statistical differences *(p* < 0.05) between black walnut (*n* = 7), maple (*n*= 4), and sycamore (*n* = 8) syrup types.

**Table 2:** Color values and browning intensity of walnut, maple, and sycamore syrups.

Sample Code	Tree Type	L*	a*	b*	Browning Intesity (BI)	
WS 1	Walnut	17.79 ± 0.03	0.36 ± 0.06	0.75 ± 0.09	0.357	0.229 ± 0.116 ^AB^
WS 2	Walnut	18.04 ± 0.04	2.01 ± 0.09	1.10 ± 0.03	0.141	
WS 3	Walnut	17.60 ± 0.34	0.54 ± 0.13	0.71 ± 0.05	0.403	
WS 4	Walnut	17.99 ± 0.08	1.27 ± 0.09	1.05 ± 0.02	0.163	
WS 5	Walnut	18.09 ± 0.05	0.66 ± 0.02	1.16 ± 0.03	0.27	
WS 6	Walnut	17.90 ± 0.01	1.58 ± 0.07	0.87 ± 0.06	0.104	
WS 7	Walnut	17.88 ± 0.02	0.70 ± 0.04	0.80 ± 0.08	0.165	
MS 1	Maple, Golden	55.53 ± 0.03	0.19 ± 0.10	25.34 ± 1.14	0.002	0.028 ± 0.028 ^B^
MS 2	Maple, Amber	19.51 ± 0.03	8.43 ± 0.03	3.62 ± 0.03	0.058	
MS 3	Maple, Amber	41.27 ± 0.02	11.29 ± 0.05	36.16 ± 0.03	0.023	
MS 4	Maple, Dark	27.13 ± 0.02	17.8 ± 0.03	16.48 ± 0.04	0.030	
SS 1	Sycamore	22.18 ± 0.14	16.17 ± 0.11	8.27 ± 0.10	0.109	0.417 ± 0.341 ^A^
SS 2	Sycamore	24.22 ± 0.09	17.56 ± 0.11	11.73 ± 0.11	0.153	
SS 3	Sycamore	17.77 ± 0.05	0.72 ± 0.02	0.80 ± 0.04	0.875	
SS 4	Sycamore	17.75 ± 0.03	0.52 ± 0.06	0.82 ± 0.06	0.737	
SS 5	Sycamore	17.80 ± 0.05	0.47 ± 0.06	0.96 ± 0.02	0.853	
SS 6	Sycamore	20.03 ± 1.27	2.60 ± 0.50	2.30 ± 0.55	0.270	
SS 7	Sycamore	19.46 ± 0.00	7.43 ± 0.19	4.37 ± 0.08	0.138	
SS 8	Sycamore	19.38 ± 1.36	1.10 ± 0.13	1.95 ± 0.49	0.20	

Values are expressed as mean ± standard deviations of triplicate replications. L*: lightness, a*: redness, b*: yellowness. Different letters (A–B) in the BI column indicate significant statistical differences (*p* < 0.05) in the browning intensity between walnut (*n* = 7), maple (*n* = 4), and sycamore (*n* = 8) syrup types.

**Table 3:** Antimicrobial activities of collected syrups.

Syrup Type		*E. coli*	*P. aeruginosa*	*S. aureus*
Black walnut syrups	WS 1	-	+	-
	WS 2	-	-	-
	WS 3	-	-	+++
	WS 4	-	+	-
	WS 5	-	-	+++
	WS 6	-	-	-
	WS 7	-	+	-
Maple syrups	MS 1	-	-	-
	MS 2	-	-	-
	MS 3	-	-	-
	MS 4	-	-	-
Sycamore syrups	SS 1	-	+	+++
	SS 2	-	-	+
	SS 3	-	+++	+++
	SS 4	+	+++	+++
	SS 5	+++	+	+++
	SS 6	-	+++	+++
	SS 7	+	+	+++
	SS 8	+	+++	+++

Inhibition zone: +++: 0.8–1.5cm, ++: <0.8cm, +: weak (thin layer), -: no activity.

## Data Availability

The original contributions presented in the study are included in the article and Supplementary Material; further inquiries can be directed to the corresponding author.

## Supplementary Materials

The following supporting information can be downloaded at: https://www.mdpi.com/article/10.3390/foods13172780/s1, Table S1: Phenolic acid and flavonoid compounds and their relative abundance in maple, black walnut, and sycamore syrups.
