# Supplementary material for: Physicochemical and Functional Properties of Black Walnut and Sycamore Syrups
Source: Foods. 2024 Aug 31;13(17):2780. doi: 10.3390/foods13172780 (PMC11395506; doi:10.3390/foods13172780)
Supplement: Supplementary file 1 [file foods-13-02780-s001.zip › foods-3180061-supplementary.pdf]

Table S1. Phenolic acid and flavonoid compounds and their relative abundance in maple, black walnut, and sycamore syrups.

Name	Formula	Classification	Calc. MW	m/z	RT [min]	Intensity (peak area) 10^6			Relative abundance*		
						Maple	Sycamore	Walnut	Maple	Sycamore	Walnut
Mesaconic acid	$C_5H_6O_4$	Phenolic Acid	130.02638	129.0191	0.05	2.6	34.7	9.8	7.5	100.0	28.3
4-Decylbenzenesulfonic acid	$C_{16}H_{26}O_3S$	Phenolic Acid	298.16173	297.1545	1.28	878.6	21.6	8.0	100.0	2.5	0.9
2-Sulfobenzoic acid	$C_7H_6O_5S$	Phenolic Acid	201.99436	200.9871	1.48	25.3	3547.3	5.1	0.7	100.0	0.1
1,3-Phenylenedioxy-diacetic Acid	$C_{10}H_{10}O_6$	Phenolic Acid	226.04884	225.0416	1.50	18.6	5.9	6.4	100.0	31.5	34.6
3,5-Dihydroxybenzoic acid	$C_7H_6O_4$	Phenolic Acid	154.02585	153.0186	1.54	127.9	122.4	59.3	100.0	95.7	46.3
5-Oxohexanoic acid	$C_6H_{10}O_3$	Phenolic Acid	130.0627	129.0554	1.55	72.5	15.7	34.7	100.0	21.7	47.9
Methylmalonic acid	$C_4H_6O_4$	Phenolic Acid	118.02617	117.0189	1.55	2.6	0.4	58.1	4.5	0.7	100.0
Pestapyrone B	$C_{11}H_{10}O_6$	Flavonoid	238.04774	237.0405	1.56	16.7	34.8	9.7	48.0	100.0	27.9
Phenylpyruvic Acid	$C_9H_8O_3$	Phenolic Acid	164.04663	163.0394	1.56	773.2	30.1	6.1	100.0	3.9	0.8
Homogentisic Acid	$C_9H_{12}O_3$	Phenolic Acid	168.07791	167.0706	1.56	3.3	15.0	3.4	22.1	100.0	22.5
6-formylsalicylic acid	$C_8H_6O_4$	Phenolic Acid	166.02603	165.0186	1.57	6.7	57.7	29.7	11.7	100.0	51.5
Chalconaringenin 2'-xyloside	$C_{20}H_{20}O_9$	Flavonoid	404.11217	403.1049	1.57	0.6	21.6	91.1	0.7	23.7	100.0
Phthalic acid	$C_8H_6O_4$	Phenolic Acid	166.02683	165.0196	1.58	34.4	25.3	132.6	25.9	19.1	100.0
4-Hydroxybenzoic acid	$C_7H_6O_3$	Phenolic Acid	138.03143	137.0242	1.59	156.5	0.4	30.9	100.0	0.3	19.8
Acetylsalicylic acid	$C_9H_8O_4$	Phenolic Acid	180.04256	179.0353	1.59	15.8	600.7	11.5	2.6	100.0	1.9

3-O-2'-methylbutyl-4a,10a-dihydrofusarubin A	$C_{20}H_{26}O_7$	Flavonoid	378.16817	377.1609	1.59	27.1	0.9	0.4	100.0	3.5	1.6
Phenylglyoxylic acid	$C_8H_6O_3$	Phenolic Acid	150.0316	149.0243	1.60	56345.3	757.6	0.2	100.0	1.3	0.0
2,5-Dihydroxyterephthalic acid	$C_8H_6O_6$	Phenolic Acid	198.01695	197.0097	1.60	6.4	9.0	3.6	71.6	100.0	40.6
3-Benzoylpropionic acid	$C_{10}H_{10}O_3$	Phenolic Acid	178.06334	177.0561	1.60	34.7	600.7	18.6	5.8	100.0	3.1
Phenoxyacetic acid	$C_8H_8O_3$	Phenolic Acid	152.04736	151.0401	1.60	23.9	8.3	97.6	24.5	8.5	100.0
3-Phenylpropanoic acid	$C_9H_{10}O_2$	Phenolic Acid	150.06803	149.0608	1.61	414.7	17.3	35.6	100.0	4.2	8.6
Phthalonic acid	$C_9H_6O_5$	Phenolic Acid	194.02199	193.0147	1.61	23.0	34.7	0.5	66.3	100.0	1.4
2-Methyl-1,2,3-propanetricarboxylic acid	$C_7H_{10}O_6$	Phenolic Acid	190.04825	189.041	1.84	72.5	9.7	200.0	36.2	4.9	100.0
3,5-Dioxocyclohexanecarboxylic acid	$C_7H_8O_4$	Phenolic Acid	156.04218	155.0349	1.85	62626.9	28202.6	5087.5	100.0	45.0	8.1
5,7,3',5'-Tetrahydroxy-3,6,8,4'-tetramethoxyflavone	$C_{19}H_{18}O_{10}$	Flavonoid	406.09142	405.0841	1.85	381.4	0.4	24.6	100.0	0.1	6.5
6-C-Xylosylluteolin	$C_{20}H_{18}O_{10}$	Flavonoid	418.09166	417.0844	1.87	70.3	72.5	6.4	97.0	100.0	8.9
Cortalcerone	$C_6H_6O_4$	Phenolic Acid	142.02611	141.0185	1.88	5.6	26.2	14.7	21.4	100.0	56.1
3-phenyllactic acid	$C_9H_{10}O_3$	Phenolic Acid	166.06222	165.0549	1.89	178.0	37.9	20.2	100.0	21.3	11.4
Methanesulfonylacetic Acid	$C_3H_6O_4S$	Phenolic Acid	137.99835	136.9911	1.89	7.5	757.6	0.9	1.0	100.0	0.1
DL-Malic acid	$C_4H_6O_5$	Phenolic Acid	134.02121	133.0139	1.90	53.2	409.7	34.7	13.0	100.0	8.5
Syringic Acid	$C_9H_{10}O_5$	Phenolic Acid	198.05274	197.0451	1.91	9.9	69.4	34.5	14.3	100.0	49.7

4-Hydroxy-3-nitrophenylacetic acid	$C_8H_7NO_5$	Phenolic Acid	197.03302	196.0257	1.92	57.3	70.3	53.2	81.6	100.0	75.8
3_4-Dihydroxymandelate	$C_8H_8O_5$	Phenolic Acid	184.03684	229.0354	1.92	20.3	11.5	37.0	54.9	31.2	100.0
Chrysin 7-glucuronide	$C_{21}H_{18}O_{10}$	Flavonoid	430.09191	429.0846	1.93	600.7	108.6	0.4	100.0	18.1	0.1
4-Hydroxybenzoyl 6-Deoxy-α-L-talopyranoside	$C_{13}H_{16}O_7$	Flavonoid	284.08977	283.0826	1.93	11.7	58.0	17.9	20.2	100.0	30.9
Phaseolorin B	$C_{15}H_{16}O_7$	Flavonoid	308.08991	367.1038	1.97	18.1	81.8	20.7	22.2	100.0	25.3
2-(3-Amino-4-chlorobenzoyl)benzoic acid	$C_{14}H_{10}ClN O_3$	Phenolic Acid	275.03398	274.0267	1.99	11.5	5.9	1.3	100.0	50.7	11.6
1,3,4,5-Tetrahydroxycyclohexanecarboxylic acid	$C_7H_{12}O_6$	Phenolic Acid	192.06371	191.0564	2.39	1.3	26.4	1.8	5.1	100.0	6.7
trans-Aconitic acid	$C_6H_6O_6$	Phenolic Acid	174.01673	173.0095	3.19	7.5	26.1	38.6	19.4	67.7	100.0
isopropylmalic acid	$C_7H_{12}O_5$	Phenolic Acid	176.06879	175.0615	3.22	757.6	600.7	3.4	100.0	79.3	0.4
2-Furoic acid	$C_5H_4O_3$	Phenolic Acid	112.01559	111.0083	3.30	15.3	19.7	32.5	47.2	60.7	100.0
Pipecolic acid	$C_6H_{11}NO_2$	Phenolic Acid	129.07868	128.0714	6.86	757.6	15.3	409.7	100.0	2.0	54.1
Citraconic acid	$C_5H_6O_4$	Phenolic Acid	130.02635	129.0191	7.25	2.6	687.3	15.7	0.4	100.0	2.3
Vanillyl mandelic acid	$C_9H_{10}O_5$	Phenolic Acid	198.05322	197.0459	7.43	7.5	2.6	409.7	1.8	0.6	100.0
3,4,5-trihydroxycyclohex-1-ene-1-carboxylic acid	$C_7H_{10}O_5$	Phenolic Acid	174.05298	173.0457	7.45	330.7	3547.3	0.9	9.3	100.0	0.0
D-(-)-Quinic acid	$C_7H_{12}O_6$	Phenolic Acid	192.06293	191.0557	8.02	547.3	551.8	130.8	99.2	100.0	23.7
5-Oxo-6-(sulfanylmethyl)-1-thia-4-azacyclotridecane-3-carboxylic acid	$C_{13}H_{23}NO_3S_2$	Phenolic Acid	305.11287	304.1056	8.76	34.7	41.6	2.0	83.4	100.0	4.9
Isovanillylmandelic Acid	$C_9H_{10}O_5$	Phenolic Acid	198.05241	197.0451	10.51	0.6	30.9	6.3	2.0	100.0	20.2

Carriebowlinol	$C_{10}H_{12}ClNO$	Flavonoid	197.06112	196.0539	10.71	24.6	3215.7	20.8	0.8	100.0	0.6
3-Methoxy-4-hydroxyphenylglycolglucuronide	$C_{15}H_{20}O_{10}$	Flavonoid	360.10557	359.0983	11.31	16.6	108.6	5.8	15.2	100.0	5.3
Flavoroseoside	$C_{15}H_{18}O_{9}$	Flavonoid	342.09535	341.0881	11.96	0.4	34.9	0.6	1.2	100.0	1.8
Ethyl-β-D-glucuronide	$C_{8}H_{14}O_{7}$	Phenolic Acid	222.07364	221.0664	13.30	54.1	405.2	135.5	13.4	100.0	33.5
Glucopyranuronic Acid	$C_{6}H_{10}O_{7}$	Phenolic Acid	194.04224	193.0349	13.31	20.2	757.6	357.1	2.7	100.0	47.1
N-{6-[(2-Carboxy-4,6-dinitrophenyl)amino]hexanoyl}-D-glucopyranosylamine	$C_{19}H_{26}N_{4}O_{12}$	Flavonoid	502.15356	501.1463	15.14	24.7	21.3	14.4	100.0	86.2	58.1
1-O-(2-aminobenzoyl)-α-L-rhamnoside	$C_{13}H_{17}NO_{6}$	Flavonoid	283.10555	284.1128	7.87	1.8	44.0	47.2	3.8	93.3	100.0
2',4'-Dihydroxy-2''- (1-hydroxy-1-methylethyl) dihydrofuro [2,3-h] flavanone	$C_{20}H_{20}O_{6}$	Flavonoid	356.1256	357.1329	2.18	17.1	4.3	1.1	100.0	25.1	6.6
4-Hydroxy-2',4'-dimethoxydihydrochalcone	$C_{17}H_{18}O_{4}$	Flavonoid	286.12036	287.1276	1.57	15.0	4.9	1.4	100.0	32.8	9.0
5,7,2',3',4'-Pentamethoxyflavanone	$C_{20}H_{22}O_{7}$	Flavonoid	374.13617	375.1435	1.87	25.5	11.7	4.7	100.0	45.7	18.6
5,7,4′-trihydroxyisoflavone-7-O-(4″-O-methyl)-β-d-glucopyranoside	$C_{22}H_{22}O_{10}$	Flavonoid	446.12084	447.1281	1.96	1.4	23.4	0.6	6.0	100.0	2.7
Astropyrone	$C_{19}H_{22}O_{5}$	Flavonoid	330.14656	331.1539	1.86	113.4	7.7	2.3	100.0	6.8	2.1
Cinnamaldehyde	$C_{9}H_{8}O$	Flavonoid	132.05783	133.0651	12.96	11.5	24.0	10.5	48.0	100.0	43.7
Coleophomone E	$C_{20}H_{22}O_{5}$	Flavonoid	342.14662	343.1539	1.57	18.5	4.1	6.1	100.0	22.3	32.8
Desmosdumotin C	$C_{19}H_{20}O_{4}$	Flavonoid	312.13597	313.1433	1.59	25.3	4.7	1.5	100.0	18.7	5.8
Diploquinone A	$C_{12}H_{10}O_{5}$	Flavonoid	234.05271	235.06	1.59	1.8	29.5	6.4	6.0	100.0	21.8
Erythro-1-Phenylpropane-1,2-diol	$C_{9}H_{12}O_{2}$	Phenolic Acid	152.08384	153.0911	1.58	10.5	19.1	7.0	55.0	100.0	36.5
Flavoroseoside	$C_{15}H_{18}O_{9}$	Flavonoid	342.0949	343.1022	8.82	1.6	78.2	8.8	2.0	100.0	11.2

Helquinoline	$C_{12}H_{15}NO_3$	Phenolic Acid	221.10512	222.1124	1.57	3.9	8.2	20.7	18.6	39.7	100.0
Hypnum acid	$C_{22}H_{16}O_9$	Flavonoid	424.07869	425.086	10.08	3.9	0.4	20.9	18.9	2.0	100.0
Isoliquiritigenin 4-methyl ether	$C_{16}H_{14}O_4$	Flavonoid	270.08912	271.0964	1.88	79.3	19.4	27.0	100.0	24.5	34.0
Oxyclozanide	$C_1H_6C_{15}NO_3$	Flavonoid	398.87771	200.4461	18.72	0.2	11.2	0.2	2.0	100.0	2.0
Pterofuran	$C_{16}H_{14}O_5$	Flavonoid	286.08409	287.0914	1.57	2.3	13.6	3.1	17.0	100.0	23.2
Pygmeine	$C_{11}H_{15}N_3O_4$	Phenolic Acid	253.10615	254.1134	14.82	7.0	6.5	1051.6	0.7	0.6	100.0
Similanpyrone C	$C_{19}H_{20}O_6$	Flavonoid	344.12575	345.133	2.17	31.9	13.9	2.2	100.0	43.7	7.0
Spiroxin D	$C_{20}H_{12}O_7$	Flavonoid	364.05837	365.0656	8.74	5.9	42.5	36.0	13.9	100.0	84.6
Triangularin	$C_{17}H_{16}O_4$	Flavonoid	284.10465	285.1119	1.86	24.1	3.7	1.7	100.0	15.5	7.1
Vitexin 2"-O- (2"'-methylbutyryl)	$C_{26}H_{28}O_{11}$	Flavonoid	516.16291	517.1702	1.92	0.5	30.3	0.8	1.5	100.0	2.8

*Relative abundance was determined using pooled qualitative reference samples of maple, black walnut, and sycamore syrups (equal amounts of each), and was calculated by dividing the peak area intensity of each syrup by the intensity of the highest peak among all syrups.
